# Enantioselective Effects of Metalaxyl Enantiomers on Breast Cancer Cells Metabolic Profiling Using HPLC-QTOF-Based Metabolomics

**DOI:** 10.3390/ijms18010142

**Published:** 2017-01-12

**Authors:** Ping Zhang, Wentao Zhu, Dezhen Wang, Jin Yan, Yao Wang, Lin He

**Affiliations:** 1 College of Plant Protection, Southwest University, Chongqing 400715, China; pingz@swu.edu.cn; 2 Beijing Advanced Innovation Center for Food Nutrition and Human Health, Department of Applied Chemistry, China Agricultural University, Beijing 100193, China; wentaozhu@cau.edu.cn (W.Z.); wangdezhen@cau.edu.cn (D.W.); ping17028@gmail.com (J.Y.); yaow@cau.edu.cn (Y.W.)

**Keywords:** metalaxyl, metabolomics, enantiomers, HPLC-QTOF, metabolic perturbations

## Abstract

In this study, an integrative high-performance liquid chromatography coupled with quadrupole time-of-flight tandem mass spectrometry (HPLC-QTOF) based metabolomics approach was performed to evaluate the enantioselective metabolic perturbations in MCF-7 cells after treatment with *R*-metalaxyl and *S*-metalaxyl, respectively. Untargeted metabolomics profile, multivariate pattern recognition, metabolites identification, and pathway analysis were determined after metalaxyl enantiomer exposure. Principal component analysis (PCA) and partitial least-squares discriminant analysis (PLS-DA) directly reflected the enantioselective metabolic perturbations induced by metalaxyl enantiomers. On the basis of multivariate statistical results, a total of 49 metabolites including carbohydrates, amino acids, nucleotides, fatty acids, organic acids, phospholipids, indoles, derivatives, etc. were found to be the most significantly changed metabolites and metabolic fluctuations caused by the same concentration of *R*-metalaxyl and *S*-metalaxyl were enantioselective. Pathway analysis indicated that *R*-metalaxyl and *S*-metalaxyl mainly affected the 7 and 10 pathways in MCF-7 cells, respectively, implying the perturbed pathways induced by metalaxyl enantiomers were also enantioselective. Furthermore, the significantly perturbed metabolic pathways were highly related to energy metabolism, amino acid metabolism, lipid metabolism, and antioxidant defense. Such results provide more specific insights into the enantioselective metabolic effects of chiral pesticides in breast cancer progression, reveal the underlying mechanisms, and provide available data for the health risk assessments of chiral environmental pollutants at the molecular level.

## 1. Introduction

The extensive application of pesticides in agriculture, individual households, and in public spaces leaves pervasive residue in the environment and exposes both humans and animals to direct and indirect sources, including via food consumption; lawn, garden, and household use; and occupational exposure [1,2]. Several pesticides are known to be associated with cancer development in experimental and epidemiological studies of farmers as well as pesticide manufacturing workers [3,4,5,6]. Although the mechanisms of cancer development induced by pesticide exposure are not clear, some potential mechanisms are DNA damage, immune response abnormality, oxidative stress chronic inflammation, and chromosome aberration [7,8,9,10]. Given the widespread applications and pervasive residue associated with pesticides, there is a need to be vigilant in surveillance of potential cancer impacts on humans.

**Figure 1:**
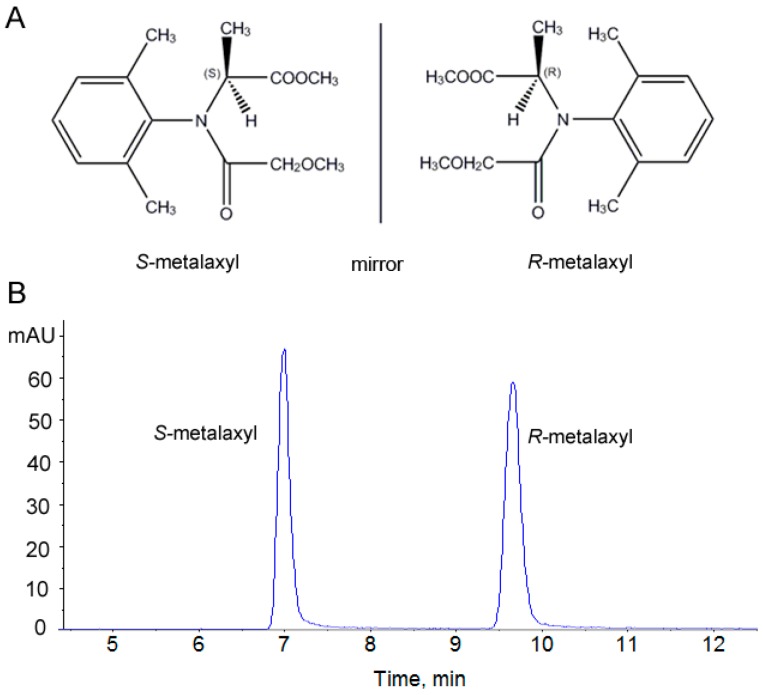
Chemical structure (**A**) and representative chromatogram (**B**) of metalaxyl enantiomers.

Metalaxyl [*N*-(2,6-dimethylphenyl)-*N*-(methoxyacetyl)-d,l-alaninemethylester, 57837-19-1] is a systemic fungicide with curative actions against oomycetes and water mold fungi in plants [11]. Its effectiveness comes from specific inhibition of RNA polymerase-1 activity and uridine incorporation into RNA process [12]. Given its broad spectrum of activity, metalaxyl is not only used on food crops but also on non-food and residential crops including tobacco, trees, ornamental plants, and lawns. Metalaxyl has an asymmetric carbon atom and consists of two enantiomers, which possess similar physicochemical properties in non-chiral environments and different activities in biological systems (Figure 1) [13]. Although metalaxyl is classified as a low-toxicity pesticide, it is mobile, persistent, and readily leached in soils. Metalaxyl residue was detected in groundwater, which poses a great threat to human and animal health [9]. Studies demonstrated cytogenetic effects of metalaxyl on human and animal chromosomes in vitro [14]. Furthermore, cocarcinogenic potential and nephrotoxicity were also reported in mice [15,16].

Breast cancer is one of the most common cancers in women and the American Cancer Society estimates that in 2016 breast cancer will have accounted for 29% of all new cancer cases diagnosed in women in the USA [17,18]. It is widely recognized that the development of cancer is the combined results of genetic predisposition and environmental factors. There is considerable evidence to show that cumulative and sustained estrogen exposure is a key promoter of breast tumor proliferation [19]. Although metalaxyl is not classified as an estrogen disrupter, studies have demonstrated that it has cytotoxicity, nephrotoxicity, cell transformation, and cocarcinogenic activity in mice [20,21,22]. Furthermore, metalaxyl has the ability to activate human pregnane X receptor, which is highly related to breast cancer cell metabolism [23]. Human pregnane X receptor (hPXR) is a primary transcription factor of CYP3A4 and an efflux transporter of multi-drug resistance gene (MDR1). Activation of hPXR will lead to upregulated expression of CYP3A4 and MDR1, which are two possible mediators of hPXR-related drug resistance in breast cancer [24]. Thus metalaxyl exposure may significantly affect the metabolome of MCF-7 cells through activating hPXR.

To further understand biological systems’ response to metalaxyl enantiomers exposure, a metabolomics profile was adopted to create a full picture of cell metabolic perturbations. Metabolomics is an important branch in the area of “omics” research, which is defined as the systematic study of multiparametric metabolic responses of organisms to perturbations such as diseases, environmental factors, genetic variations, and other stimuli [25,26,27]. Over the past decades, there has been an increased interest in cellular metabolism, which is regarded as a possible means of cancer treatment, especially after the vital finding of upregulated glucose consumption and lactate production in cancer cells under aerobic conditions by Otto Warburg in the 1930s [28]. Altered metabolism helps cancer cells sustain a higher proliferation rate even in suboptimal environments, resist certain cell signals, and also prevent immune response. Therefore, alterations in cellular metabolism are expected as one of the vital hallmarks in cancer development [29]. Some major metabolic alterations including enhanced fatty acids synthesis, upregulated utilization of the pentose phosphate pathway, increased production of lactate, altered utilization of the tricarboxylic acid (TCA) cycle, and reduced transport of pyruvate into mitochondria were identified in cancer cells [30]. Therefore, the emerging field of metabolomics may provide insights into integrated perturbed metabolic profile in cancer research and help us find key biomarkers, which may act as a reliable clinical tool for cancer diagnostic screening and therapy [31].

The aim of this study was to evaluate the enantioselective metabolic perturbations induced by metalaxyl enantiomers in MCF-7 cells using a high-performance liquid chromatography coupled with quadrupole time-of-flight tandem mass spectrometry (HPLC-QTOF) based untargeted metabolomics approach. HPLC-QTOF based metabolomics profile, multivariate pattern recognition, metabolites identification, and pathway analysis were determined in MCF-7 cells after R-metalaxyl and S-metalaxyl treatments, respectively. To our best knowledge, this is the first enantioselective study of metabolic alterations induced by metalaxyl enantiomers in breast cancer cells using metabolomics techniques. Such results may contribute to explaining the enantioselective metabolic and toxic effects of chiral pesticides in breast cancer progression, revealing the underlying mechanisms, and providing available data for health risk assessments of chiral environmental pollutants.

## 2. Results and Discussion

### 2.1. Assessment of the Stability and Reproducibility of the HPLC-QTOF Method

Quality control (QC) samples were obtained by pooling metalaxyl enantiomer-treated cell samples and prepared using the same protocol as sample preparation. For the stability of HPLC-QTOF system, QC sample was injected randomly during the sequence analysis. As for the reproducibility of the sample preparation, 1000 µL of a mixed control sample was split into five parts with 200 µL each and treated with the same preparation protocol. A blank sample (ultrapure water), prepared in the same way as the other samples, was injected every five samples to minimize the carry-over between sample analyses.

The results for system stability and preparation method reproducibility are shown in Figure S1. We examined coefficient of variation (CV) values of 1061 mass variables that were detected in 70% of QC data. As shown in Figure S1, more than 90% of QTOF variables had CV <20%, and <4% had CV >30%, indicating excellent system stability. For reproducibility, more than 80% of QTOF variables had CV <20%, and <7% had CV >30%, which also implied good reproducibility. Such results indicate that the established analytical method is highly stable and reproducible, and could be employed to analyze large-scale cell samples in metabolomics experiments.

### 2.2. Multivariate Pattern Recognition Analysis

**Figure 2:**
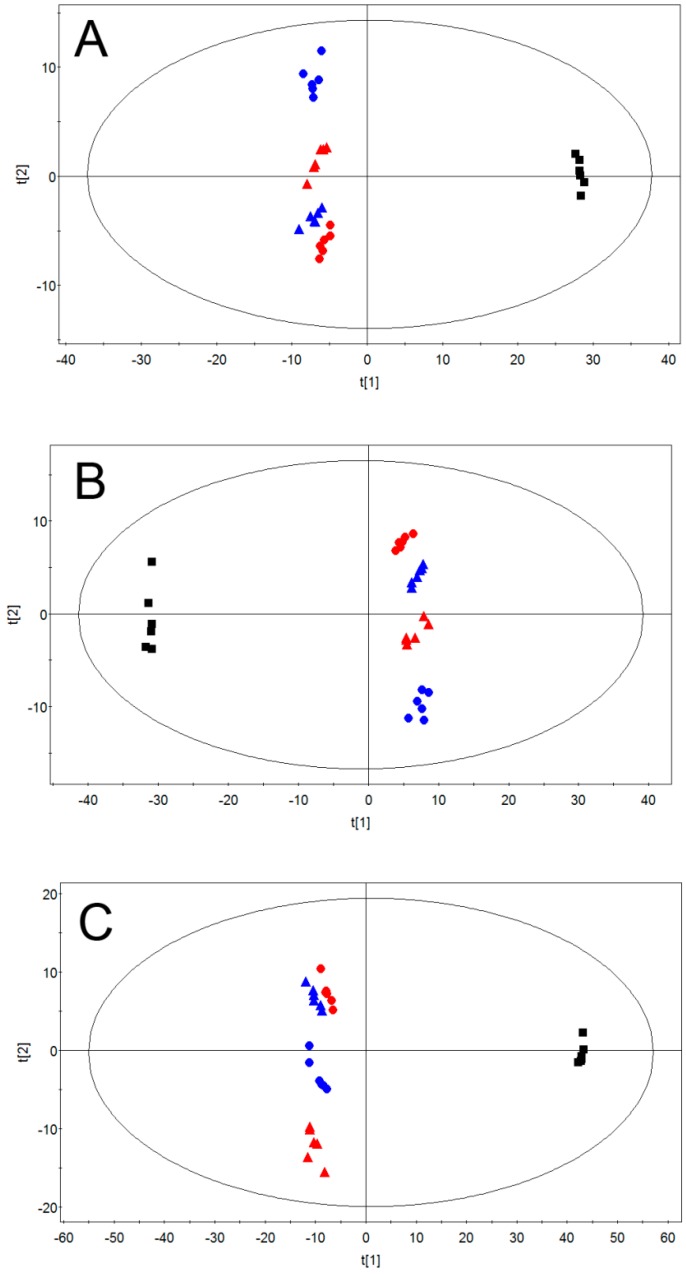
PCA score plots based on QTOF data sets of MCF-7 cells treated with different concentrations of metalaxyl enantiomers. ■ Control, ▲ *S*-metalaxyl (10 µM), ▲ *S*-metalaxyl (50 µM), ● *R*-metalaxyl (10 µM), ● *R*-metalaxyl (50 µM). (**A**) Positive mode, (*R*^2^*X* = 0.656, *Q*^2^ = 0.552); (**B**) negative mode, (*R*^2^*X* = 0.715, *Q*^2^ = 0.629); (**C**) negative + positive mode, (*R*^2^*X* = 0.783, *Q*^2^ = 0.685).

**Figure 3:**
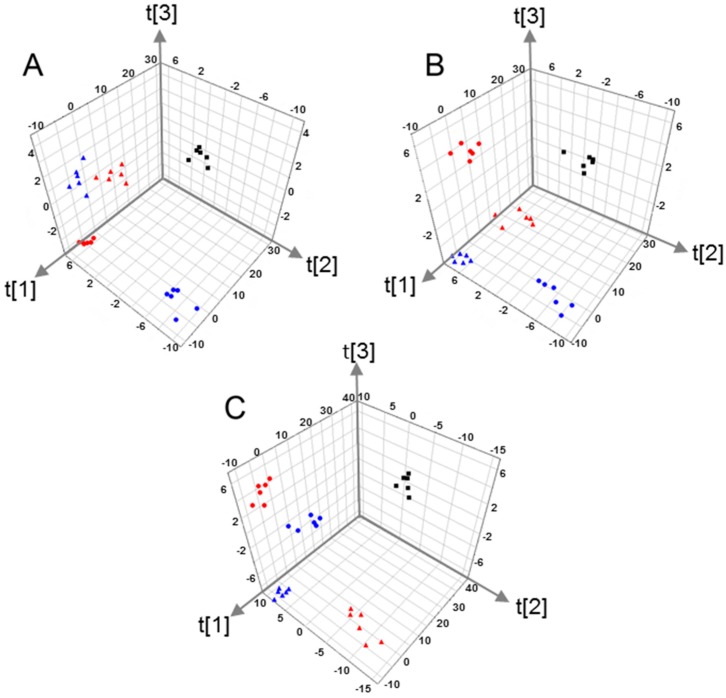
The PLS-DA score plots based on QTOF data sets of MCF-7 cells treated with different concentration of metalaxyl enantiomers. ■ Control, ▲ *S*-metalaxyl (10 µM), ▲ *S*-metalaxyl (50 µM), ● *R*-metalaxyl (10 µM), ● *R*-metalaxyl (50 µM). (**A**) Positive mode, (*R*^2^*X* = 0.829, *R*^2^*Y* = 0.982, *Q*^2^ = 0.913); (**B**) negative mode, (*R*^2^*X* = 0.816, *R*^2^*Y* = 0.99, *Q*^2^ = 0.957); (**C**) negative + positive mode, (*R*^2^*X* = 0.803, *R*^2^*Y* = 0.986, *Q*^2^ = 0.948).

In order to investigate the global metabolites changes in MCF-7 cells after metalaxyl enantiomer exposure, Principal component analysis (PCA) was employed to analyze the QTOF data set first. PCA is an unsupervised multivariate data analysis method often adopted to visualize grouping trends and outliers in data. Principle component 1 (PC1) versus component 2 (PC1) of MCF-7 cells in positive, negative, and both modes are shown in Figure 2. Good separation between metalaxyl enantiomer-treated groups and the control group were displayed in PC1 and PC2 plots of PCA, suggesting that metalaxyl enantiomers significantly disturbed the MCF-7 cell metabolome after seven days of consecutive exposure. The fitness and prediction capabilities of PCA modes were evaluated by *R*^2^*X*(cum) and *Q*^2^(cum), respectively. Figure 2 also showed *Q*^2^*X*(cum) and *Q*^2^(cum) of PCA in different modes. *Q*^2^ in the positive, negative, and both modes were all over 0.5, displaying the excellent predictive capability. In addition, metabolome changes induced by *R*-metalaxyl and *S*-metalaxyl were also clearly separated, which means *R*-metalaxyl and *S*-metalaxyl have enantioselective effects on MCF-7 cell metabolite profiling. Moreover, cells treated with a single enantiomer at different concentrations (10 and 50 µM) were clearly separated, indicating that the metabolome change attributable to different treated concentrations was also different. To further identify the metabolites that account for the PCA separation, supervised PLS-DA was employed to construct the mode based on HPLC-QTOF ion peak areas of cell metabolites. Representative PLS-DA plots of treated groups and the control group are shown in Figure 3. *R*-metalaxyl- and *S*-metalaxyl-treated groups are clearly separated with the control group. Moreover, the *R*-metalaxyl-treated group and *S*-metalaxyl-treated group also displayed a clear separation, which means the metabolite changes induced by the two enantiomers were enantioselective. Generally, *R*^2^*X*, *R*^2^*Y*, and *Q*^2^*Y* were adopted to assess the quality of the PLS-DA mode. Without a high *R*^2^*Y*, it is impossible to obtain a high *Q*^2^*Y* and a robust model was linked to a *Q*^2^ > 0.4 [32]. As shown in Figure 3, the parameters of *R*^2^*X*, *R*^2^*Y*, and *Q*^2^*Y* were acceptable for single negative and positive mode datasets, and the combination of two datasets also displayed an excellent prediction, with *R*^2^*Y* = 0.986 and *Q*^2^*Y* = 0.948.

### 2.3. Metabolite Identification

**Figure 4:**
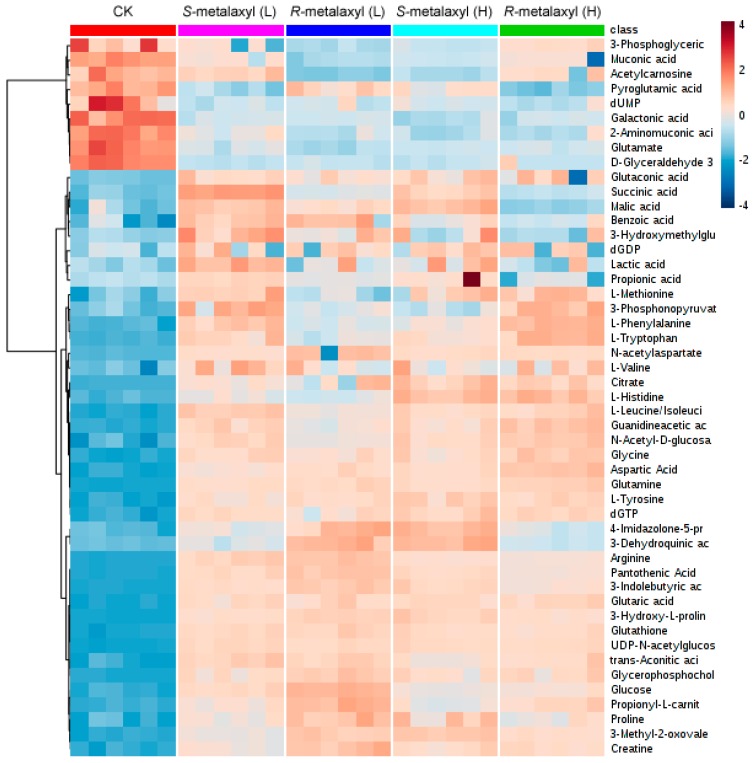
Heat map produced by the most significantly differential metabolites.

To determine the metabolites that are responsible for metalaxyl enantiomer intervention, each *R*-metalaxyl- and *S*-metalaxyl-treated group was compared with a control group by PLS-DA analysis. The variable importance in the project (VIP) value derived from the PLS-DA mode is an important parameter for each independent variable. Higher VIP scores are considered more relevant in classification. In this study, the VIP value of each peak was calculated to identify its contribution to the classification. On the basis of the VIP threshold (VIP > 1) and the student’s *t* test *p* value (*p* < 0.05), a total of 49 metabolites (35 from the negative mode and 14 from the positive mode) were finally identified (Table 1). These significant changed metabolites were amino acids, nucleotides, fatty acids, carbohydrates, phospholipids, indoles, derivatives, and so on. *R*-metalaxyl- and *S*-metalaxyl-treated (10 µM) groups induced 27 and 36 endogenous metabolite increases, and five and eight metabolites decreased, respectively. High concentration (50 µM) caused 29 and 35 endogenous metabolites to be upregulated, and six and eight metabolites to be downregulated, respectively. Such results indicated that metabolic profiling changes caused by the same concentration of *R*-metalaxyl and *S*-metalaxyl were enantioselective. Furthermore, some metabolites changed with the treated concentration, such as histidine, lactate, glucose, alanine, succinate, citrate, and arginine (Figure 4).

### 2.4. Biological Pathway Analysis

**Figure 5:**
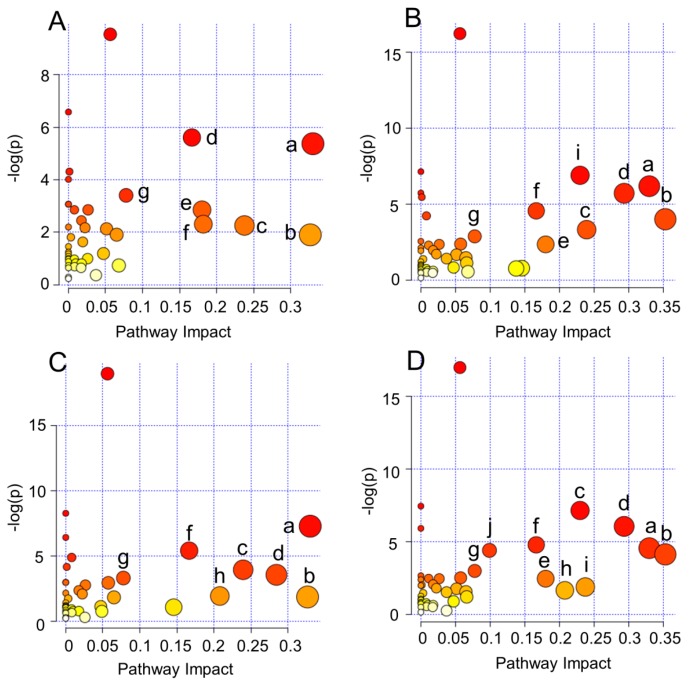
Global metabolic pathways affected by metalaxyl enantiomers based on MetaboAnalyst 3.0. (**A**) *S*-metalaxyl (10 µM); (**B**) *R*-metalaxyl (10 µM); (**C**) *S*-metalaxyl (50 µM); (**D**) *R*-metalaxyl (50 µM). a. Glycine, serine, and threonine metabolism; b. d-Glutamine and d-glutamate metabolism; c. Glutathione metabolism; d. Arginine and proline metabolism; e. Pantothenate and CoA biosynthesis; f. Phenylalanine metabolism; g. -TCA cycle-; h. Histidine metabolism; i. Alanine, aspartate, and glutamate metabolism; j. Phenylalanine, tyrosine, and tryptophan biosynthesis.

**Figure 6:**
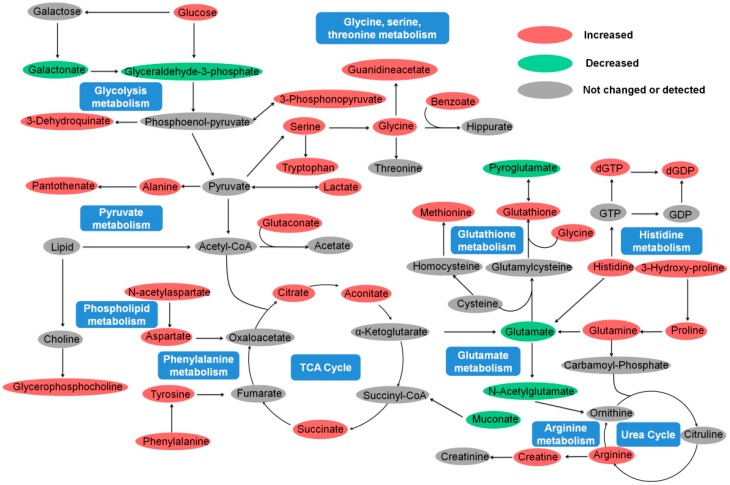
Perturbed pathways and fluctuant metabolites in MCF-7 cells induced by metalaxyl enantiomers.

Metabolomics profiling can reveal not only the individual metabolite alteration but also provide a comprehensive view of the metabolic processes induced by toxic environmental compounds. In this study, metalaxyl enantiomer-induced metabolic perturbations were further evaluated at the metabolic pathways level on the basis of these different metabolites. The MetaboAnalyst 3.0 was employed to reveal the most significantly affected pathways induced by metalaxyl enantiomers. *R*-metalaxyl mainly affected seven pathways, including Glycine, serine and threonine metabolism, d-Glutamine and d-glutamate metabolism, Glutathione metabolism, Pantothenate and CoA biosynthesis, Arginine and proline metabolism, -TCA cycle- and Phenylalanine metabolism. Whereas, *S*-metalaxyl mainly caused 10 pathway perturbations, including Glycine, serine and threonine metabolism, d-Glutamine and d-glutamate metabolism, Glutathione metabolism, Alanine, aspartate and glutamate metabolism, Histidine metabolism, Arginine and proline metabolism, Phenylalanine, tyrosine and tryptophan biosynthesis, Pantothenate and CoA biosynthesis, -TCA cycle-and Phenylalanine metabolism. Such results implied that the pathways perturbed by metalaxyl enantiomers were also enantioselective. In addition, Figure 5 indicates that metabolic pathways changes caused by the same concentration of *R*-metalaxyl and *S*-metalaxyl are also enantioselective. Such perturbed pathways are highly related to energy metabolism, amino acid metabolism, lipid metabolism, and antioxidant defense (Figure 6).

### 2.5. Amino Acid Metabolism

Amino acids and their metabolites are critical to life and play a variety of roles in metabolism. Besides their roles as building blocks of polypeptides and proteins, some amino acids including proline, arginine, glutamine, tryptophan, cysteine, and leucine are critical for growth, maintenance, immunity, and reproduction in organisms. Lots of studies also indicate that the abnormal metabolism of amino acids impairs development and growth, perturbs whole body homeostasis, and even causes death. Thus, physiological concentrations of amino acids and their metabolites are relatively constant and highly regulated at the molecular level. In the present study, cell metabolomics results revealed that amino acid metabolism were significantly perturbed due to metalaxyl enantiomers exposure, including Glycine, serine and threonine metabolism, Alanine, aspartate and glutamate metabolism, d-Glutamine and d-glutamate metabolism, Phenylalanine metabolism, Arginine and proline metabolism, Tryrosine metabolism, and so on. Metalaxyl caused increased levels of alanine, tyrosine, phenylalanine, serine, tryptophan, glycine, proline, glutamine, arginine, methionine and decreased levels of glutamate, and *N*-acetylglutamate. These amino acids regulate specific pathways and have different functions in cell metabolism. Much evidence also shows that amino acids including alanine, glutamine, glutamate, lysine, phenylalanine, and glycine also participate in specific cell metabolism, cell signaling, and oxidative stress [33,34,35].

Glycine, produced in the mitochondria, has been demonstrated as an indicator of cancer cell proliferation in a previous investigation [36]. In this study, the concentration of glycine increased after metalaxyl enantiomer treatments, which implies that metalaxyl enantiomers may promote MCF-7 cell proliferation. Alanine can regulate gluconeogenesis to ensure glucose production through inhibiting l-type pyruvate kinase [37,38]. Moreover, alanine is also a known product of glucose and glutamine in cancer cells associated with β-nicotinamide adenine dinucleotide phosphate (NADPH) production. Therefore, increased levels of alanine and glucose may imply upregulated gluconeogenesis and glycolysis in MCF-7 cells after metalaxyl exposure. In cancer cells, glutamine can be utilized for the production of lactate and act as a carbon source and an amino acid for protein synthesis. Once taken up by the cell, much of the glutamine is generally converted to glutamate. It is interesting to point out that metalaxyl enantiomer treatment in MCF-7 cells leads to an increase in glutamine and a decrease in glutamate, which may be caused by upregulated glutamine synthesis and downregulated glutamine metabolism. To sum up, metalaxyl enantiomers can significantly perturb amino acid metabolism in MCF-7 cells in an enantiomer-specific way.

### 2.6. Energy Metabolism

Significant changes of many metabolites involved in energy metabolism, including glucose, lactate, alanine, citrate, aconitate, and succinate, were observed in MCF-7 cells after metalaxyl enantiomer exposure. These metabolites are highly related to energy metabolism pathways such as the TCA cycle, pyruvate metabolism, and glycolysis metabolism. The prominent perturbations of energy metabolism induced by metalaxyl enantiomers were increased levels of TCA cycle intermediates, including citrate, aconitate, and succinate in MCF-7 cells. Citrate, aconitate, and succinate are the main intermediates of the TCA cycle and play important roles in energy metabolism. Although other factors cannot be excluded, the increased levels of citrate, aconitate, and succinate indicated that the activities of mitochondrial enzymes involved in the TCA cycle were significantly affected in MCF-7 cells after metalaxyl enantiomer exposure, resulting in the fluctuation of energy metabolism. Furthermore, the increased levels of citrate and aconitate induced by *R*-metalaxyl and *S*-metalaxyl were almost the same, while succinate increased about 1.2-fold and 1.8-fold after *R*-metalaxyl and *S*-metalaxyl exposure, respectively. This notion suggests that *R*-metalaxyl and *S*-metalaxyl have enantioselective effects on TCA cycle.

In addition, the levels of pantothenate, lactate, and alanine, which are highly related to pyruvate metabolism, were all increased. Pyruvate metabolism has been reported as one of the major alterations in breast cancer cells. Rather than importing pyruvate into mitochondria, cancer cells generally convert pyruvate into lactate. The key factor affecting pyruvate metabolism is pyruvate dehydrogenase (PDH), which is highly regulated by pyruvate dehydrogenase kinase (PDK). Inhibition of PDH will decrease transportation of pyruvate into the mitochondria through its oxidation into acetyl-CoA and lead to an increased level of lactate in the cytoplasm [30]. Therefore, the increased level of lactate may be caused by the inhibition of PDH through activating PDK, resulting in decreased transportation of pyruvate into the mitochondria and enhanced conversion of pyruvate to lactate in MCF-7 cells. Furthermore, some gluconeogenic amino acids, including phenylalanine, histidine, methionine, glutamine, tyrosine, and alanine, were all increased, which may imply promoted gluconeogenesis after metalaxyl exposure.

We also found an increased level of creatine in MCF-7 cells after metalaxyl enantiomers exposure. The major function of creatine is to support energy production in the phosphorylation process. Interestingly, studies reported that creatinine and creatine kinase were considered oxidative stress indicators in breast cancer patients after chemotherapy treatment [39]. Therefore, the upregulated level of creatine clearly implies the alteration of oxidative stress and energy metabolism in MCF-7 cells after metalaxyl enantiomer exposure. Taking all these results together, metalaxyl enantiomers significantly perturbed energy metabolism in MCF-7 cells and the degrees affected by two enantiomers were enantioselective.

### 2.7. Lipid Metabolism and Antioxidant Defense

Based on metabolomics results, the upregulated glycerophosphocholine and related amino acids clearly reflected the perturbation of lipid metabolism after metalaxyl enantiomer exposure. Glycerophosphocholine (GPC) is known to be an important endogenous compound required for cell and mitochondrial membranes, neurotransmitter synthesis, methylation-dependent biosynthesis, lipid transportation, and bile acid secretion [40,41,42]. In addition, GPC is a vital constituent of the cell membrane and lipoprotein phospholipid, which play important roles in the integrity of the cell membrane and lipid metabolism. Evidence shows that GPC metabolite deficiency contributes to various disorders in humans, including fatty liver development, liver steatosis, hepatocarcinogenesis, and mitochondrial dysfunction [43,44,45]. GPC can not only sustain choline and its metabolites’ homeostasis but also protects cells and their organelles from oxidative stress and inflammation [46,47,48,49]. In this study, the level of GPC increased after *R*-metalaxyl and *S*-metalaxyl exposure, possibly induced by the high demand of membrane synthesis for cell proliferation after metalaxyl enantiomer exposure.

Another interesting finding is the increased level of glutathione (GSH) after metalaxyl exposure. As the most abundant thiol-containing compound in the cell, GSH plays vital roles in intracellular signaling and antioxidant defense. Total GSH concentration varies significantly in different cell types and can be regulated by external factors including heavy metals, glucose concentration, and exposure to reactive oxygen species. Cancer cells are often protected from excessive damage by reactive oxygen species (ROS) through the simultaneous upregulation of innate protective antioxidant pathways and the generation of reduced glutathione [50]. As a result, the increased GSH induced by metalaxyl enantiomers clearly suggests the enhancement of antioxidant defense in MCF-7 cells, which may be beneficial to promote proliferation of breast cancer cells.

## 3. Materials and Methods

### 3.1. Chemicals and Materials

Racemic metalaxyl (*rac*-metalaxyl) standard (purity ≥ 98.0%) was obtained from the Institute for Control of Agrochemicals, China Ministry of Agriculture (Beijing, China). *S*-metalaxyl and *R*-metalaxyl were prepared via high-performance liquid chromatography (HPLC) (Agilent, Santa Clara, CA, USA) with a semi-preparative chiral column containing cellulose-*tris*-(3,5-methylphenylcarbamate)-based chiral stationary phase (CDMPC-CSP). Water was purified with a Milli-Q Element system from Millipore (Billerica, MA, USA). HPLC grade methanol, acetonitrile, ammonium acetate, and acetic acid were purchased from Merck Chemicals (Beijing, China). All other chemicals and solvents were analytical grade and purchased from commercial sources.

### 3.2. Cell Culture Procedures and Metabolite Extraction

The MCF-7 cell line was purchased from the China Center for Type Culture Collection (CCTCC) and cultured in DMEM High Glucose supplemented with 2 mM glutamine, 25 mM glucose, 100 g/mL penicillin/streptomycin, and 10% fetal bovine serum (FBS). Cells were seeded onto 150mm plates and the medium was changed every second day until the confluency reached 80%, at which time they were washed with PBS and incubated in DMEM medium containing metalaxyl enantiomers for seven consecutive days. The new medium with metalaxyl enantiomers was also changed every second day. Two concentrations (10 and 50 µM) were selected for *S*-metalaxyl and *R*-metalaxyl treatments separately and seven replicate samples were collected for each treatment. After washing the cells with PBS three times, the number of living cells was counted using a Neubauer counting chamber under a light microscope and protein content was analyzed by the kits at the end point. Cold extraction solvent methanol:choloroform (9:1) was added to quench the cellular metabolism. The cells were detached using a cell scraper and the cell suspensions were transferred into tubes and centrifuged. Finally, the supernatants were collected and dried under a vacuum using Eppendorf Concentrator plus (Eppendorf, Germany). Then they were reconstituted in 500 µL 60% acetonitrile/40% water solvent containing 5 mM ammonium acetate and 0.2% acetic acid. All samples were purified with 0.22 µm filters before HPLC-QTOF analysis.

### 3.3. HPLC-QTOF Analysis

The HPLC-QTOF analysis was conducted on an Agilent 1200 series HPLC system coupled with an electrospray ionization (ESI) source and Agilent 6510 QTOF mass spectrometry (Agilent, USA). In all cases, 10 µL of extracted sample was injected into a reversed-phase column (ACQUITY BEH C_18_, 150 mm × 2.1 mm, 1.7 µm, Waters, Milford, CT, USA) equipped with a guard cartridge system. The flow rate was 0.3 mL/min and the column temperature was maintained at 40 °C. The mobile phase was composed of solvents A (10% acetonitrile/90% H_2_O containing 5 mM ammonium acetate and 0.2% acetic acid) and B (90% acetonitrile/10% H_2_O containing 5 mM ammonium acetate and 0.2% acetic acid). The gradient conditions for both modes were identical and are shown in Table S1 in the Supplementary Materials.

Data were collected in the positive and negative electrospray modes in separate runs with full scan mode ranges from *m*/*z* 60 to 1000. The capillary voltages were 3800 and −4000 V for positive mode and negative mode, respectively, with a scan rate of 1.03 scans per second. The nebulizer gas flow rate was 10 L/min; the pressure was maintained at 45 psi and the temperature at 325 °C. Reference masses 121.0509, 922.0098 (positive mode) and 119.0363, 966.0007 (negative mode) were used for continuous and online mass calibration throughout the analyses. All samples were injected in one randomized sequence and kept in the LC auto-sampler maintained at 4 °C.

### 3.4. Data Treatment

The resulting data files were cleaned of unrelated ions and background noise by the Molecular Feature Extractor (MFE) tool in the MassHunter Qualitative Analysis Software (Agilent, USA). The MFE is a compound-finding technique that can extract individual compound features from QTOF-MS chromatogram even when chromatograms are complicated and compounds are not well resolved. Lastly, MFE can output a list of all possible compound features extracted from full scan QTOF data. The MassHunter Mass Profiler Professional Software B.02.00 (Agilent, USA) was used to align and filter off extracted features. We selected metabolites with absolute abundance over 5000 counts and with a minimum of two ions. Metabolites from different samples were aligned using a retention time window of 0.1% (0.15 min) and multiple charge states were not selected. Common features represented in at least 80% of all samples were analyzed and corrected for individual bias.

### 3.5. Biological Pathway Analysis

The major perturbed biological pathways were analyzed based on the significantly changed metabolites in MCF-7 cells after metalaxyl enantiomer exposure. In this study, metabolic pathway perturbations were conducted by MetaboAnalyst 3.0 (http://www.metaboanalyst.ca/) and related metabolic pathway profiles were determined based on the Kyoto Encyclopedia of Genes and Genomes (KEGG) pathway database (http://www.kegg.jp/kegg/pathway.html).

### 3.6. Statistical Analysis

Metabolite differences between metalaxyl-treated cell samples and control samples were evaluated using student’s *t* test analysis. Multivariate data analysis was conducted with the SIMCA-P software package (V11.0, Umetrics, Sweden). Principal component analysis (PCA) was conducted based on QTOF datasets to generate an overview for group clustering and to search for possible outliers. Partial least-squares discriminant analysis (PLS-DA) was employed to explore the significant changed metabolites and group clustering based on QTOF datasets. Significantly changed metabolites were identified based on student’s *t* test (with *p* < 0.05) and variable influence on project score (VIP > 1). Accurate masses of significant changed features were further identified by Mass Profiler Professional Software (Agilent, USA) and also confirmed against HMDB (http://www.hmdb.ca), METLIN (https://metlin.scripps.edu), KEGG (http://www.kegg.jp), and LIPID MAPS (http://www.lipidmaps.org) database.

## 4. Conclusions

In the present study, the enantioselective metabolic perturbations in MCF-7 cells induced by metalaxyl enantiomers were evaluated using HPLC-QTOF-based metabolomics. Our results indicated that metabolic profiles of MCF-7 cells were significantly altered in both metalaxyl enantiomer-treated groups compared with control group. Furthermore, the endogenous metabolite changes and pathway fluctuations induced by metalaxyl enantiomers were enantioselective. HPLC-QTOF-based metabolomics demonstrated that metalaxyl enantiomers mainly disrupted amino acid metabolism, energy metabolism, lipid metabolism, and antioxidant defense. Our study illustrates that QTOF-based metabolomics is very sensitive and suitable for monitoring the systematic metabolic effects of metalaxyl enantiomer exposure in MCF-7 cells, which can provide integrative information for the enantioselective effects of chiral pesticides in breast cancer progression, reveal the underlying mechanisms, and provide available data for the health risk assessment of chiral environmental pollutants at the molecular level.

## Figures and Tables

**Table 1:** Significant changed metabolites induced by *R*-metalaxyl and *S*-metalaxyl in MCF-7 cells. C, Control group; L, Low-dose group; H, High-dose group.

No.	Mode	Compounds	Mass	*S*-Metalaxyl L vs. C			*S*-Metalaxyl H vs. C			*R*-Metalaxyl L vs. C			*R*-Metalaxyl H vs. C			Class
				VIP ^a^	FC ^b^	Trend ^c^	VIP	FC	Trend	VIP	FC	Trend	VIP	FC	Trend	
1	Negative	Lactate	72.0213	1.14	3.55	↑	<1	2.80	↑	<1	2.28	↑	<1	1.66	↑	Hydroxy acids and derivatives
2	Negative	Propionate	74.0368	1.17	1.92	↑	<1	2.22	↑	1.13	1.42	↑	1.09	1.38	↑	Carboxylic acids and derivatives
3	Negative	Glycine	75.0322	1.18	3.64	↑	1.16	3.55	↑	1.17	3.44	↑	1.19	3.91	↑	Carboxylic acids and derivatives
4	Negative	Alanine	89.0474	1.17	2.19	↑	1.16	2.53	↑	1.17	2.30	↑	1.19	2.72	↑	Carboxylic acids and derivatives
5	Negative	Serine	105.0420	1.15	1.62	↑	1.13	1.62	↑	1.11	1.49	↑	1.17	1.73	↑	Carboxylic acids and derivatives
6	Negative	Proline	115.0624	1.23	1.21	↑	1.57	1.26	↑	<1	1.29	↑	1.06	1.20	↑	Carboxylic acids and derivatives
7	Negative	Guanidineacetate	117.0419	1.18	2.55	↑	1.16	2.45	↑	1.18	2.22	↑	1.19	2.75	↑	Carboxylic acids and derivatives
8	Negative	Succinate	118.0259	1.18	1.85	↑	1.15	1.60	↑	1.12	1.34	↑	1.08	1.24	↑	Carboxylic acids and derivatives
9	Negative	Glutaconate	130.0256	1.16	2.11	↑	1.12	2.12	↑	1.15	1.96	↑	1.12	2.17	↑	Carboxylic acids and derivatives
10	Negative	3-Methyl-2-oxovalerate	130.0623	<1	3.10	↑	1.03	4.01	↑	<1	3.93	↑	<1	3.53	↑	Keto acids and derivatives
11	Negative	Creatine	131.0582	1.17	2.76	↑	1.16	3.03	↑	1.17	3.45	↑	1.19	3.05	↑	Carboxylic acids and derivatives
12	Negative	Aspartate	133.0371	1.21	2.14	↑	1.23	2.24	↑	<1	2.25	↑	<1	2.47	↑	Carboxylic acids and derivatives
13	Negative	Muconate	142.0237	3.50	0.82	↓	4.12	0.75	↓	4.9	0.70	↓	4.1	0.76	↓	Fatty acids and conjugates
14	Negative	Glutamine	146.0685	1.66	4.84	↑	1.62	4.87	↑	<1	5.10	↑	<1	5.34	↑	Carboxylic acids and derivatives
15	Negative	Glutamate	147.0524	1.14	0.67	↓	1.14	0.65	↓	1.15	0.59	↓	1.16	0.66	↓	Carboxylic acids and derivatives
16	Negative	Methionine	149.0503	1.12	2.52	↑	1.01	2.32	↑	<1	1.56	↑	1.12	2.54	↑	Carboxylic acids and derivatives
17	Negative	4-Imidazolone-5-propionate	156.0523	1.15	3.19	↑	1.16	4.79	↑	1.14	4.65	↑	1.14	2.84	↑	Imidazoles
18	Negative	2-Aminomuconate	157.0353	1.12	0.58	↓	1.13	0.42	↓	1.14	0.46	↓	1.14	0.44	↓	Carboxylic acids and derivatives
19	Negative	Benzoate	157.9976	4.06	1.81	↑	3.71	1.72	↑	3.65	1.80	↑	3.39	1.71	↑	Benzene and substituted derivatives
20	Negative	3-Phosphonopyruvate	167.9816	1.10	1.88	↑	<1	1.33	↑	1.06	1.42	↑	1.05	1.87	↑	Organic phosphoric acids and derivatives
21	Negative	Glyceraldehyde-3-phosphate	169.9970	1.14	0.05	↓	1.13	0.08	↓	1.15	0.09	↓	1.13	0.14	↓	Carbohydrates and carbohydrate conjugates
22	Negative	Aconitate	174.0157	1.94	1.93	↑	1.25	1.77	↑	<1	1.91	↑	<1	1.86	↑	Carboxylic acids and derivatives
23	Negative	*N*-acetylaspartate	175.0478	2.36	3.78	↑	2.35	3.93	↑	1.28	4.62	↑	1.39	3.94	↑	Carboxylic acids and derivatives
24	Negative	Glucose	180.0629	1.98	2.15	↑	1.71	1.92	↑	1.11	2.45	↑	<1	2.01	↑	Carbohydrates and carbohydrate conjugates
25	Negative	Tyrosine	181.0728	1.18	2.68	↑	1.16	2.97	↑	1.17	2.80	↑	1.18	2.86	↑	Phenylpropanoic acids
26	Negative	*N*-Acetyl-l-glutamate	189.0632	1.76	0.11	↓	1.71	0.13	↓	<1	0.09	↓	<1	0.16	↓	Carboxylic acids and derivatives
27	Negative	3-Dehydroquinate	190.0404	<1	2.64	↑	1.22	4.38	↑	<1	4.33	↑	<1	2.37	↑	Alcohols and polyols
28	Negative	Citrate	192.0268	3.26	7.09	↑	3.56	8.97	↑	2.31	6.23	↑	2.98	8.37	↑	Carboxylic acids and derivatives
29	Negative	Galactonate	196.0571	1.14	0.24	↓	1.12	0.14	↓	1.13	0.24	↓	1.08	0.25	↓	Hydroxy acids and derivatives
30	Negative	Pantothenate	219.1099	2.04	11.52	↑	1.97	11.39	↑	1.07	13.02	↑	<1	10.04	↑	Carboxylic acids and derivatives
31	Negative	*N*-Acetyl-d-glucosamine	257.0662	1.07	2.29	↑	1.03	2.27	↑	<1	2.01	↑	<1	2.41	↑	Carbohydrates and carbohydrate conjugates
32	Negative	Acetylcarnosine	306.0308	<1	0.95	↓	1.66	0.82	↓	<1	0.77	↓	<1	0.91	↓	Carboxylic acids and derivatives
33	Negative	dUMP	344.0442	1.24	0.73	↓	1.1	0.76	↓	<1	0.76	↓	<1	0.72	↓	Pyrimidine nucleotides
34	Negative	dGDP	427.0289	1.12	3.95	↑	1.11	4.07	↑	<1	4.16	↑	<1	3.91	↑	Purine nucleotides
35	Negative	dGTP	506.9954	1.95	2.51	↑	1.95	2.60	↑	<1	2.37	↑	<1	2.56	↑	Purine nucleotides
36	Positive	Valine	117.0779	1.27	1.57	↑	1.15	1.43	↑	1.23	1.41	↑	1.06	1.50	↑	Carboxylic acids and derivatives
37	Positive	Pyroglutamate	129.0416	1.08	0.64	↓	<1	0.81	↓	<1	0.87	↓	1.11	0.56	↓	Carboxylic acids and derivatives
38	Positive	3-Hydroxy-l-proline	131.0677	1.28	7.63	↑	1.41	8.39	↑	1.41	8.44	↑	1.26	7.58	↑	Carboxylic acids and derivatives
39	Positive	Leucine/Isoleucine	131.0932	1.82	2.98	↑	1.72	2.78	↑	1.33	2.48	↑	1.81	2.87	↑	Carboxylic acids and derivatives
40	Positive	Glutarate	132.0521	1.07	3.59	↑	1.07	3.61	↑	1.04	3.60	↑	1.11	3.68	↑	Carboxylic acids and derivatives
41	Positive	Histidine	155.0674	<1	3.47	↑	1.04	4.96	↑	<1	3.07	↑	1.06	4.94	↑	Carboxylic acids and derivatives
42	Positive	Phenylalanine	165.0773	1.43	2.05	↑	1.23	1.77	↑	1.07	1.61	↑	1.56	2.18	↑	Phenylpropanoic acids
43	Positive	Arginine	174.1099	2.13	6.52	↑	1.86	5.83	↑	2.3	6.99	↑	1.75	5.55	↑	Carboxylic acids and derivatives
44	Positive	3-Indolebutyrate	203.1137	1.75	5.98	↑	1.79	6.25	↑	1.83	6.68	↑	1.7	5.51	↑	Indoles and derivatives
45	Positive	Tryptophan	204.0874	1.07	2.41	↑	<1	2.23	↑	<1	1.91	↑	1.2	2.68	↑	Indoles and derivatives
46	Positive	Propionyl-l-carnitine	217.1288	<1	1.50	↑	<1	1.44	↑	1.1	1.72	↑	1.02	1.57	↑	Fatty acid esters
47	Positive	Glycerophosphocholine	257.1003	1.98	10.24	↑	1.98	10.24	↑	2.29	11.71	↑	2.08	10.77	↑	Glycerophospholipids
48	Positive	Glutathione	307.0812	3.54	1.98	↑	3.49	1.97	↑	3.63	2.01	↑	3.36	1.94	↑	Carboxylic acids and derivatives
49	Positive	UDP-*N*-acetylglucosamine	607.0766	1.25	4.12	↑	1.23	4.07	↑	1.21	4.03	↑	1.21	4.04	↑	Pyrimidine nucleotides

^a^ VIP represents variable importance in the project value derived from PLS-DA mode; ^b^ FC represents fold change; ^c^ ↑ represents up-regulated trend and ↓ represents down-regulated trend.

## Supplementary Materials

Supplementary materials can be found at www.mdpi.com/1422-0067/18/1/142/s1.
