# Supplementary material for: Enantioselective Effects of Metalaxyl Enantiomers on Breast Cancer Cells Metabolic Profiling Using HPLC-QTOF-Based Metabolomics
Source: Int J Mol Sci. 2017 Jan 12;18(1):142. doi: 10.3390/ijms18010142 (PMC5297775; doi:10.3390/ijms18010142)
Supplement: Supplementary file 1 [file ijms-18-00142-s001.pdf]

*Int. J. Mol. Sci.* **2017**, *18*, 142; doi:10.3390/ijms18010142 

# Supplementary Materials: Enantioselective Effects of Metalaxyl Enantiomers on Breast Cancer Cells Metabolic Profiling Using HPLC-QTOF-Based Metabolomics

**Ping Zhang, Wentao Zhu, Dezhen Wang, Jin Yan, Yao Wang and Lin He**

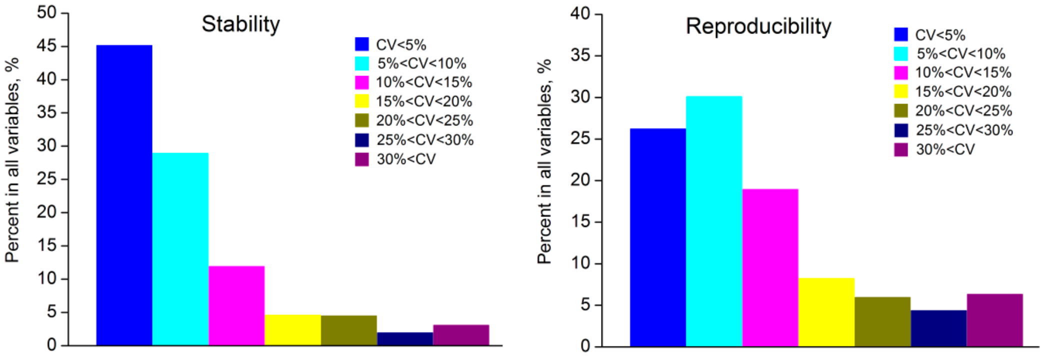


**Figure S1.** The stability and reproducibility of high-performance liquid chromatography coupled with quadrupole time-of-flight tandem mass spectrometry (HPLC-QTOF) method.

**Table S1.** The gradient of high-performance liquid chromatography coupled with quadrupole time-of-flight tandem mass spectrometry (HPLC-QTOF) separation.

| Time (min) | Flow Rate (μL/min) | Mobile Phase A | Mobile Phase B |
|---|---|---|---|
| 0 | 300 | 10 | 90 |
| 1.5 | 300 | 10 | 90 |
| 5 | 300 | 50 | 50 |
| 12 | 300 | 50 | 50 |
| 14 | 300 | 10 | 90 |
| 20 | 300 | 10 | 90 |
| 30 | 300 | 10 | 90 |
